# Comparison of Linear and Non-linear Regression Analysis to Determine Pulmonary Pressure in Hyperthyroidism

**DOI:** 10.12669/pjms.331.11046

**Published:** 2017

**Authors:** Camelia C. Scarneciu, Livia Sangeorzan, Horatiu Rus, Vlad D. Scarneciu, Mihai S. Varciu, Oana Andreescu, Ioan Scarneciu

**Affiliations:** 1 Camelia C. Scarneciu, MD, PhD. Faculty of Medicine, Transilvania University of Brasov, Brasov, Romania; 2 Livia Sangeorzan, PhD. Faculty of Mathematics and Computer Science, Transilvania University of Brasov, Brasov, Romania; 3 Horatiu Rus, MD, PhD. Faculty of Medicine, Transilvania University of Brasov, Brasov, Romania; 4 Vlad D. Scarneciu, MD, PhD. Candidate. Faculty of Medicine, Transilvania University of Brasov, Brasov, Romania; 5 Mihai S. Varciu, MD, PhD. Faculty of Medicine, Transilvania University of Brasov, Brasov, Romania; 6 Oana Andreescu, MD, PhD. Faculty of Medicine, Transilvania University of Brasov, Brasov, Romania; 7 Ioan Scarneciu, MD, PhD. Faculty of Medicine, Transilvania University of Brasov, Brasov, Romania

**Keywords:** Hyperthyroidism, Pulmonary hypertension, Linear and Non-linear Regression

## Abstract

**Objectives:**

This study aimed at assessing the incidence of pulmonary hypertension (PH) at newly diagnosed hyperthyroid patients and at finding a simple model showing the complex functional relation between pulmonary hypertension in hyperthyroidism and the factors causing it.

**Methods:**

The 53 hyperthyroid patients (H-group) were evaluated mainly by using an echocardiographical method and compared with 35 euthyroid (E-group) and 25 healthy people (C-group). In order to identify the factors causing pulmonary hypertension the statistical method of comparing the values of arithmetical means is used. The functional relation between the two random variables (PAPs and each of the factors determining it within our research study) can be expressed by linear or non-linear function. By applying the linear regression method described by a first-degree equation the line of regression (linear model) has been determined; by applying the non-linear regression method described by a second degree equation, a parabola-type curve of regression (non-linear or polynomial model) has been determined. We made the comparison and the validation of these two models by calculating the determination coefficient (criterion 1), the comparison of residuals (criterion 2), application of AIC criterion (criterion 3) and use of F-test (criterion 4).

**Results:**

From the H-group, 47% have pulmonary hypertension completely reversible when obtaining euthyroidism. The factors causing pulmonary hypertension were identified: previously known- level of free thyroxin, pulmonary vascular resistance, cardiac output; new factors identified in this study- pretreatment period, age, systolic blood pressure. According to the four criteria and to the clinical judgment, we consider that the polynomial model (graphically parabola- type) is better than the linear one.

**Conclusions:**

The better model showing the functional relation between the pulmonary hypertension in hyperthyroidism and the factors identified in this study is given by a polynomial equation of second degree where the parabola is its graphical representation.

## INTRODUCTION

Pulmonary hypertension on hyperthyroid patients is one of the most recent and up-to-date research topics. The guidelines for diagnosis and pulmonary hypertension treatment published in 2015^1^ classify PH from hyperthyroidism belonging to group 5 of the pulmonary hypertension with unclear and/or multifactorial mechanisms. Many studies based on a small number of cases reports an increased incidence of pulmonary arterial hypertension in hyperthyroid patients.^2^-^5^

These studies show that pulmonary hypertension on hyperthyroid patients is mainly determined by the hormonal excess and the increased cardiac output (CO) and pulmonary vascular resistance (PVR). Also, the same studies suggest that autoimmunity^4^ is an important factor that causes pulmonary hypertension with increased PVR in Graves’ disease. The conclusion of the studies shows that the hormonal excess is the proper cause of pulmonary hypertension due to the fact that pulmonary hypertension appears at the same moment with hyperthyroidism and disappears when the status of euthyroidism is obtained. Instead, these coefficients of determination and correlation are small and lacking statistical significance. On the other hand, during the medical research, the evaluation of the determining relation between two variables one uses the coefficient of determination R^2^ usually calculated by using the linear regression method. This one suggests the fact that the relation of determination between two variables is either increasing or decreasing, namely, a limitless relation. For human bodies, there are some limits of viability that cannot be surpassed. Nevertheless, the relation of determination cannot be linear as physiological mechanisms of adaptation and regulation intervene and model this line.

The study, aims at assessing the incidence of pulmonary hypertension (PH) at newly diagnosed hyperthyroid patients and at finding a simple model showing the complex functional relation between pulmonary hypertension in hyperthyroidism and the factors causing it.

## METHODS

Our research study was approved by the Transilvania University of Brasov, Ethics Committee. It has taken place over a period of 12 months, in 2015/2016, on newly diagnosed hyperthyroid patients’ who presented in endocrinology ambulatory and sent for enrollment to Medical Semiology Discipline.

Inclusion criteria have consisted of clinical manifested hyperthyroidism, newly diagnosed, under the age of 50 (18-48 years), without any cardiovascular, pulmonary, autoimmune disease associated. Only 53 hyperthyroid patients signed the informed consent, and only 35 came back for follow-up appointments, after 12 weeks in euthyroid clinical status. Therefore, in this research, we used a hyperthyroid group (H-group, n=53), a euthyroid group (E-group, n=35) and a control group (C- group, n=25).

The design of the study consisted in a determination of hormonal profile, an M and 2D Echocardiography combined with a tissue and spectral Doppler followed by the installment of a 24h EKG Holter and ABMP for 24 hour. These investigations were applied to the H-group, at the moment of inclusion, after 12 weeks of anti-thyroid treatment when euthyroidism by installing (thus becoming group E), and to the C-group at the time of enrollment.

### Hormonal analysis

For hormonal analysis, we used an ARCHITECT machine (ABBOT, USA). Free thyroxin (FT_4_) was detected by chemiluminescence method with the normal value of 10-23 pmol/l.

### Echocardiographic and Doppler examination

For echographic measurements, we used a “Philips Sparq ultrasound machine”, following standard procedures. For calculating pulmonary vascular resistance (PVR), we used Lindquist formula.^6^ Transthoracic echocardiographic examinations were performed by a single experienced echocardiographer with Philips Sparq and Philips Sonos 7500 ultrasound machines (Philips, USA), and the echographic measurements (M and 2D) were made following standard procedures.^6^ Using the 3-points Simpson’s method we calculate the left ventricular ejection fraction (LVEF). The measurement of CO was made by using 2D echography and the Simpson technique, by multiplying the systolic volume and the cardiac rate. Measurements of transmitral flux and E wave peak velocity were made using 2D echography. Through tissue Doppler, we measured the E’ wave maximal velocity in proto-diastole at the medial mitral ring level. The E/E’ ratio as a marker of diastolic function of the left ventricle independent of preload was used. The pulmonary artery systolic pressure (PAPs) is calculated as the sum of the right atrium pressure and the tricuspid pressure gradient, both echographical estimates. Using Bernoulli’s modified equation and the maximum velocity of tricuspid regurgitation, we calculated the trans-tricuspid pressure gradient, by using continuous wave Doppler The pressure in the right atrium was estimated by considering the diameter and collapse of the inferior venae cavae. PVR was calculated using the Lindquist 7 formula as follows:

PVR=(PAPm-10)/CO, where: PVR = pulmonary vascular resistance, PAPm = median pressure in the pulmonary artery, CO = cardiac output.

The median pressure in the pulmonary artery was calculated using the following formula:

PAPm=0.61 x PAPs+2 mm Hg, where: PAPm= median pressure in the pulmonary artery, PAPs = systolic pressure in the pulmonary artery.

PH is defined as PAPm≥25 mm Hg at rest^1^, determined by catheterization of the right atrium. In order to estimate PAWP (pulmonary arterial wedge pressure) the tissue Doppler examination has been used. The ratio between transmitral early diastolic flow velocity (E) and early diastolic mitral annular velocity and (E ’) was calculated.

If the ratio E/E’>11 and the left ventricle ejection fraction is normal, that is more than 50% (LVEF≥50%), or if the ration E/E’>15 and the left ventricle ejection fraction is low, under 50% (LVEF<50%) then PAWP is increased.^1^ A PAPm≥25 mm Hg and a normal PAWP signify a pre-capillary pulmonary hypertension, and a PAPm≥25 mm Hg increased PAWP signify a post-capillary pulmonary hypertension.^1^

PAP can be estimated from continuous wave Doppler measurements. Echocardiography is performed when pulmonary hypertension is suspected and may be used to determine a diagnosis of PH in patients in whom multiple different echocardiographic measurements led to the same diagnosis. Echocardiography is recommended as a first-line non-invasive diagnostic investigation of PH, when suspected, first class indication evidence level C.

### EKG Holter recording for 24 h

For EKG/24 hour HOLTER recording we used a “Schiller AG, MT-100”. In this paper, we only used the means values of the heart rate/24 hours (HR/24h).

### ABPM recording for 24 hour

For ABPM/24 hours recording we used a Meditech ABPM-5 device. In this study, we utilized only mean values of systolic blood pressure/24 hours (systolic BP/24h).

### Statistical analysis

We use the program MedCalc Statistical Software version 17 (MedCalc Software bvba, Ostend, Belgium; https://www.medcalc.org; 2017). In our tables below, the measured parameters are presented as mean values±S.D. (standard deviation). We applied the t-test for the statistical significance and χ^2^ Chi-squared test to study the Normal distribution.^8^,^9^ A p value <0.05 was considered statistically significant for confidence interval (CI) of a 95% for the difference between the two arithmetic mean.

The linear^8^,^9^ or non-linear regression^9^, is used to fit data to a model that defines a functional relation between two random variables, a dependent variable y (outcome variable, PAPs in our study) and one independent variable x (predictor, FT_4_, pretreatment period, age, cardiac output, systolic blood pressure and pulmonary vascular resistance).

The functional relation between the two random variables can be linear, named a basic linear regression, described using a first degree equation y=a+bx (where *b* is the slope and *a* is a constant (intercept) of the line), relation being always graphically expressed by a line.^8^ If in the 95% CI for slope the value zero is included the model is rejected.^9^ The functional relation between the two random variables can be non-linear, also called non-linear regression (polynomial of degree 2, 3; exponential; logarithmical, etc.) and graphically represented by a regression curve. The best mathematical polynomial model describing the results of our experiments is expressed by a second degree equation y=a +bx+cx^2^ relation being always graphically expressed by a parabola.^9^

In our study we have made a comparative analysis between two types of regression starting from the point that, inside a biological system, the relations of functional determination among various variables are less probably linear.

There is always a difference between scatter plot graph resulted in the representation of measured data and the regression model represented by a line or a curve. The vertical distances from the measured points to the corresponding points (predicted) on either regression line or the regression curve are called residuals.^8^,^9^

The best model taken into consideration is that where the error (residual) sum of squares (SSE) is the least.^9^ We can say that the model fits the data in our case. An important parameter in the study of regression models is the coefficient of determination R^2^.^8^,^9^ The value of R^2^ (in terms of percentage) expresses the percentage of the variability of the outcome (PAPs in our study) that can be explained by the predictor.

Generally, for these two models, the higher value of the coefficient of determination R^2^ suggests a better model. If the residuals and R^2^ register close values for the two models, then, we can distinguish which of the two models is the most convenient consequently, a F-test (applied for nested model)^9^ or the application of AIC criterion (Akaike’s Information Criterion^9^) are required. The F-test^8^, as a test for analyzing variance, applies for each type of regression, separately. The result is expressed as the F-ratio, from which a “p value” is calculated and accepts (p<0.05) or rejects (p>0.05) each regression model taken separately.

The method AIC determines the way in which the data supports each model, taking into account both the residual-sum-of-squares and the number of parameters in the model.^9^ If the difference between the AIC of the polynomial model and the AIC of the linear model (Δ AIC) is negative then the polynomial model is accepted and is considered the best one.^9^

## RESULTS

### Study population

Mean values ±S.D. of the measured parameters, in the three groups (H, E, C), the statistical significance of the means comparison (p-value), are shown in Table-I. Hyperthyroid groups (H), consist of 53 patients, 42 with Graves’ disease, ten with multinodular goiter, one with autonomous adenoma. The debut of the disease was general with 12.4±7 weeks anterior to the diagnostic (pretreatment period). Judging by the data in Table-I, there aren’t any differences between the two groups regarding age, sex. The values of PAPs are statistically significantly increased in the H-group compared to the E- and the C- group. Worth mentioning is the fact that PAPs values for euthyroid patients are similar to those in the C-group, which suggests the fact that PH is completely reversible when obtaining euthyroidism.

**Table-I:** Study population.

Parameter	(1) H-group (n=53)	(2) C-group (n=25)	p value (1/2)	(3) E-group (n=35)	p value (2/3)
Age(years)	35.5±8.4	36.8±8.9	ns	36.2±8.4	ns
Women(%)	79.2	84	ns	77.8	ns
PAPs(mmHg)	35.7±7.3	24.2±2.7	<0.0001	23.9±2.9	ns
FT_4_(pmol/l)	59.1±24.1	15.3±2.6	<0.0001	15.6±3.1	ns
PVR(WU)	2.1±0.5	1.3±0.4	<0.0001	1.3±0.3	ns
CO(l/min)	6.6±0.8	5.2±0.5	<0.0001	5.2±0.5	ns
LVEF(%)	69.8±4.6	66.7±4.7	0.007	67.5±4.7	ns
HR/24h(beats/min)	101.7±7.5	80.2±4.1	<0.0001	81±5.6	ns
E/E’	4.9±1	4.7±0.8	ns	4.6±0.7	ns
Systolic BP/24h (mmHg)	130±14.8	106.5±4.1	<0.0001	106.8±4.3	ns

Note: H-group Hyperthyroid group; C-group Controls group; E-group Euthyroid group; PAPs-systolic pressure in the pulmonary artery; FT_4_-free thyroxin; PVR-pulmonary vascular resistance; CO-cardiac output; LVEF-left ventricular ejection fraction; HR-heart rate; E/E’ – ratio of peak velocity of early diastolic filling(E)/E’ wave maximal velocity; Systolic BP-mean systolic BP/24h; WU-Wood units; ns-not significant (p≥0.05).

The values of main determinants of PAPs, FT_4_, CO and PVR, are also increased in the hyperthyroid group, at a significant level. Left ventricular ejection fraction (LVEF) and heart rate (HR) are main determinants of CO and are raised to in the H-group, at a significant level. We also observed significantly high values of systolic BP. Similar values in the E- and C-group, for all these parameters, suggest that PVR, CO, HR, LVEF, systolic BP values normalize when obtaining euthyroidism. According to the 2013 ESH/ESC guidelines for the management of arterial hypertension, 24 hyperthyroid patients have fulfilled criteria for arterial hypertension (45.2%).

### Hyperthyroid Group with PH comparative with Hyperthyroid Group without PH

Measured parameters in the H-group with PH comparing with the ones without PH are shown in Table-II. The PH is considered at a PAPm≥25mmHg.^1^ PH was detected at 25 hyperthyroid patients (which represent 47% of the H-group) and at no one of the other two groups. PAPs≥35mmHg and an E/E’ ratio of <11, considering that LVEF>50% confirm the fact that the PH was solitarily arterial.^3^

**Table-II:** Hyperthyroid group with PH comparative with hyperthyroid group without PH.

Parameter	(1) H with PH (n=25)	(2) H without PH (n=28)	p-value (1/2)	(3) C-group (n=25)	p-value (1/3)	p value (2/3)
Age(years)	31±8.6	39.6±5.9	<0.0001	36.8±8.9	0.003	ns
Pretreatment period (weeks)	14.6±4.9	10.5±8.0	0.03			
PAPs(mmHg)	42.2±4.8	29.9±2.7	<0.0001	24.2±2.7	<0.0001	<0.0001
FT_4_(pmol/L)	57.8±22.0	60.3±26.1	ns	15.3±2.6	<0.0001	<0.0001
PVR(WU)	2.5±0.3	1.7±0.3	<0.0001	1.3±0.4	<0.0001	0.0001
CO(L/min)	7.1±0.6	6.2±0.8	<0.0001	5.2±0.5	<0.0001	<0.0001
LVEF(%)	73±2.6	67.0±4.1	<0.0001	66.7±4.7	<0.0001	ns
HR(beats/min)/24 h	103.1±8.2	100.4±6.7	ns	80.5±4.1	<0.0001	<0.0001
E/E’	4.7±1	5.1±1	ns	4.7±0.8	ns	ns
Systolic BP/24h (mmHg)	141.4±11.7	119.8±8.6	<0.0001	106.5±4.1	<0.0001	<0.0001

Note: C-group control group; PAPs-systolic pressure in the pulmonary artery; FT_4_-free thyroxin; PVR-pulmonary vascular resistance; CO-cardiac output; LVEF-left ventricular ejection fraction; HR-heart rate; E/E’ – ratio of peak velocity of early diastolic filling (E)/E’ wave maximal velocity; Systolic BP-mean systolic BP/24h; WU-Wood units: ns-not significant (p≥0.05)

The PAPs values in the H-group with PH are statistically significantly increased as compared to the H-group without PH (Table-II). In the H-group with PH, the determinants of PAPs, PVR and CO are significantly increased but in paradox, FT_4_ levelly is lower than at the patients without PH (Table-II).

The hyperthyroid patients with PH are significantly younger, have a longer pretreatment period, a higher systolic BP (92.3% have hypertension) but have Graves’ disease in similar percentage (80.7% versus 77.7%) than the hyperthyroid patients without PH. Those facts suggest that age, pretreatment period and systolic blood pressure can also be determinant for PAPs if we demonstrate the existence of a relationship between them.

### Hemodynamic parameters on age groups

The main hemodynamic characteristics of the hyperthyroid group, age groups, as mean values ±S.D, are presented in Table-III. As it can be seen in Table-III, the age group 18-28 years, at the lowest value of FT_4_, has the highest level of HR, the best LVEF, CO, PAPs, PVR and the highest systolic BP. With each decade of age, at similar levels or even increased levels of FT_4_, the PAPs, PVR, CO, LVEF and the systolic BP/24h drops.

**Table-III:** Hyperthyroid group on age groups.

Parameter	(1) 18-28years n=11	(2) 29-38years n=21	(3) 39-48years n=21	p value (1/2)	p value (1/3)	p value (2/3)
Pretreatment period (weeks)	15.8±5.6	9.7±4.6	13.4±8.6	0.002	ns	ns
PAPs(mmHg)	45.2±5.7	35.6±4.7	30.8±4.8	<0.0001	<0.0001	0.002
FT_4_(pmol/L)	63.3±19	47.7±22.8	68.3±23.9	ns	ns	0.006
PVR(WU)	2.6±0.4	2±0.4	1.9±0.5	0.0004	0.0004	ns
CO(L/min)	7.4±0.2	6.9±0.4	5.8±0.7	0.0005	<0.0001	<0.0001
LVEF(%)	75.5±0.5	70±3.3	66.7±3.9	<0.0001	<0.0001	0.005
HR(beats/min)/24 h	108.5±4	99.4±7.5	100.3±7	0.0008	0.001	ns
E/E’	5.1±1.2	5.1±1.1	4.6±0.8	ns	ns	ns
Systolic BP/24h(mmHg)	145±15.9	129.2±10.5	122.9±12.9	0.002	0.0002	ns

Note: PAPs-systolic pressure in the pulmonary artery; FT_4_-free thyroxin; PVR-pulmonary vascular resistance; CO-cardiac output; LVEF-left ventricular ejection fraction; HR-heart rate; E/E’ – ratio of peak velocity of early diastolic filling(E)/E’ wave maximal velocity; systolic BP-mean systolic BP/24h;WU-Wood units; ns-not significant(p≥0.05).

### Comparing the linear and non-linear models of determination of pulmonary pressure in hyperthyroidism

**Fig. 1:**
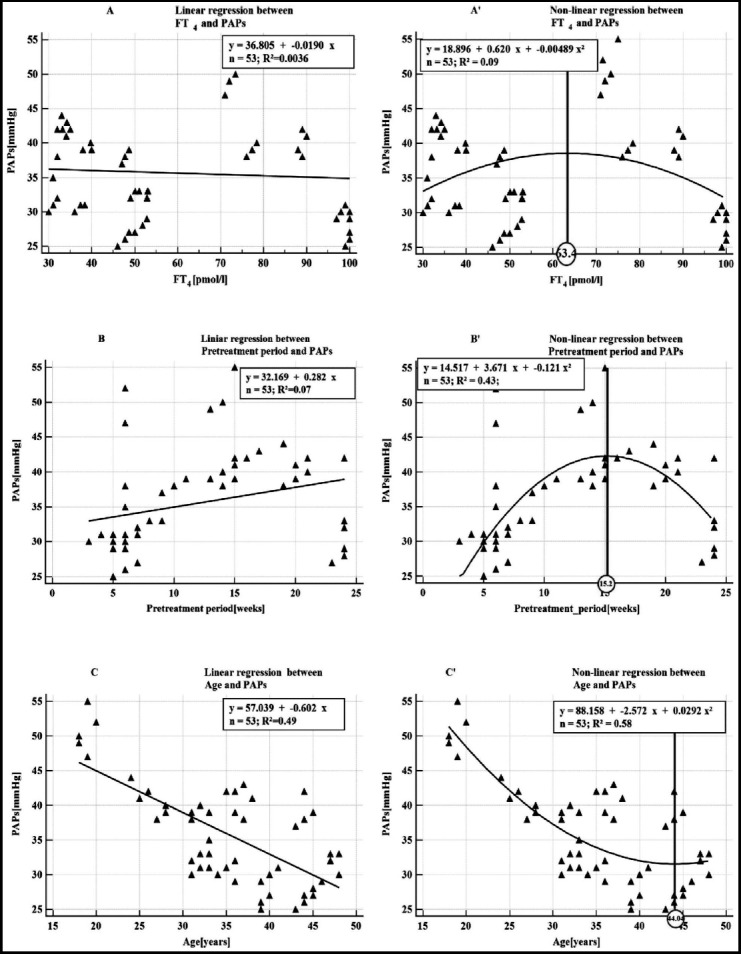
Comparison between linear and non-linear scatter diagrams of determination between PAPs and each of the variables FT4, Pretreatment period and Age

In Fig.1 the relations of determination between PAPs and each of the variables FT_4_, Pretreatment period and Age, are graphically represented by the regression line in squares A,B,C in comparison with the parabola-type regression (non-linear regression) in the squares A’, B’, C’. In the (polynomial) non-linear regression, the higher the coefficients of determination of PAPs is improved (22 times in case of FT_4_, 6 times in case of Pretreatment period and 1.2 times in case of Age) the higher level of significance of determination in comparison with the linear regression is. The parabola scatters diagram aspect is sustained by the clinical judgment. The peak of the parabola (marked with a vertical line on Fig.1 in squares A, B, C) thus highlighting the moment when counter-regulating mechanisms and adaptation becomes efficient. Thus PAPs increases at the same time with the hormonal level and the duration of the pretreatment period until the concentration of FT_4_ is about 63 pmol/l and respectively 15 weeks of disease evolution. After that PAPs will drop in spite of the increasing in hormonal levels. This behavior explains why, in studies using linear regression, the coefficients of determination between FT_4_ and PAPs are disappointing, demonstrating a rather lack of some functional connections between the two parameters. PAPs drops with age until the age of about 45 years; after which it begins to rise.

Fig.1 shows that the coefficient of determination R^2^ calculated by using the polynomial regression are higher than those calculated by using the linear regression. We can state that the polynomial model is better than the linear one.

Making the visual analysis of the plot diagram (see Fig.1), one can state that the measured plots are better approximated by the regression curve (parabola) than by the regression line.

**Fig. 2:**
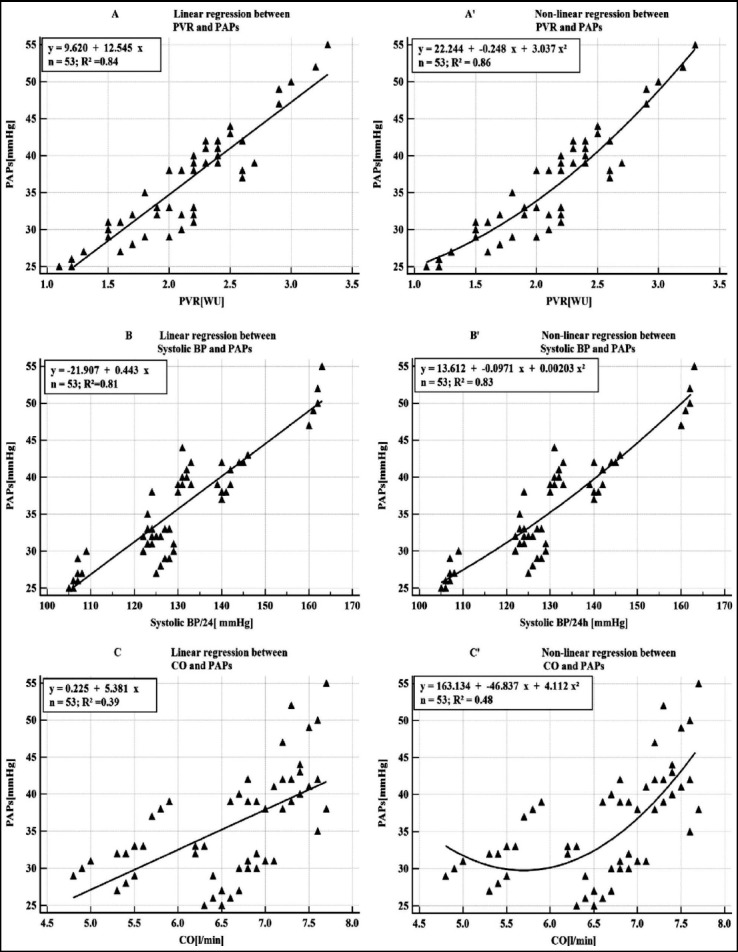
Comparison between linear and non-linear scatter diagrams of determination between PAPs and each of the variables PVR, systolic BP/24h and CO.

In Fig.2 the relations of determination between PAPs and each of the variables PVR, Systolic BP / 24h and CO, are graphically represented by the regression line in squares A,B,C in comparison with the parabola-type regression in the squares A’, B’, C’.

One can observe that both types of regression have very good coefficients of determination. However the coefficients of determination for the polynomial model are higher fact that suggests this model is considered to be better.

In Table-IV comparative analysis between the linear and polynomial models of determination between PAPs and each parameter taken separately, is made. In all cases the polynomial model is preferred as the coefficient of determination R^2^ is higher, the residual sum of squares for polynomial regression is smaller than the residual sum of squares for linear regression and ΔAIC has a negative value.^9^ Zero value in a 95% CI for slope, reject the linear regression models among PAPs and FT_4_ and PAPs and Pretreatment period.

**Table-IV:** Comparison between linear and polynomial models of determining between PAPs (outcome variable) and predictor variables.

Predictor variables	Regression Type	R^2^[1]	SSE [2]	Significance F (p<0.05) *p* value [3]	Model Validation [criterion]	ΔAIC [4]	Preferred model of regression [criterion]
FT4	Linear	0.004	2728.62	0.6533	Reject model [3]	-2.61	Polynomial [1,2,4]
	Polynomial	0.09	2501.08	0.1026	Reject model [3]		
Pretreatment period	Linear	0.07	2539.30	0.0502	Reject model [3]	-23.88	Polynomial [1,2,4]
	Polynomial	0.43	1558.26	<0.0001	Accept model [3]		
CO	Linear	0.35	1763.9	<0.0001	Accept model [3]	-9.54	Polynomial [1,2,4]
	Polynomial	0.48	1418.57	<0.0001	Accept model [3]		
Age	Linear	0.48	1411.17	<0.0001	Accept model [3]	-9.08	Polynomial [1,2,4]
	Polynomial	0.58	1144.82	<0.0001	Accept model [3]		
Systolic BP/24h	Linear	0.81	497.04	<0.0001	Accept model [3]	-0.09	Polynomial [1,2,4]
	Polynomial	0.83	477.79	<0.0001	Accept model [3]		
PVR	Linear	0.84	427.39	<0.0001	Accept model [3]	-5.39	Polynomial [1,2,4]
	Polynomial	0.86	371.74	<0.0001	Accept model [3]		

*Note*: FT_4_-free thyroxin; CO-cardiac output; systolic BP/24h-mean systolic BP/24h;PVR-pulmonary vascular resistance; R^2^-coefficient of determination;[1]-criterion 1, value of R^2^; SSE-sum of squares residual; [2]- criterion 2, value of SSE; ΔAIC-difference between AIC(Akaike’s Information Criterion) of the polynomial model and AIC of linear model; [3]- criterion 3, p value of significance of F-ratio; [4]- criterion 4, value of ΔAIC; Significance F(p<0.05)-significance level for F-ratio being a p<0.05;

By applying Fisher-test and by calculating F-ratio and F-significance the linear regression models among PAPs and FT_4_, PAPs and Pretreatment period and the polynomial regression model between PAPs and FT_4_ are rejected. Nevertheless, all the other models are accepted in all cases but the polynomial model is preferred.

## DISCUSSIONS

In the present study, the Doppler echocardiographic examination (the most common method for non-invasively estimate PAPs^7^) we reveal the presence of PH of hyperthyroidism, in 47% percent of cases, demonstrating that this is a relatively common complication in hyperthyroidism.^2^-^4^ We have also shown that PAPs normalizes when achieving euthyroidism.^2^-^4^ We conclude that hormonal excess in hyperthyroidism contributes directly to the production of PH.^3^,^4^

By using the method of polynomial regression we demonstrated PAPs increases at the same time with the hormonal level until the concentration of FT_4_ is 63,4pmol/l. Then PAPs will drop in spite of the increase in hormonal levels. This behavior explains why, in studies using linear regression, the coefficients of determination between FT_4_ and PAPs are disappointing, demonstrating a rather lack of some functional connections between the two parameter.^3^-^5^ With regard to the regression between FT_4_ and PAPs both polynomial and linear models are invalidated due to the increased value of significance F, even if the polynomial model is better than the linear one. Accepting the linear model leads to an error of 65.33%, instead, accepting the polynomial model leads to an error of 10.26%. Even if this error is lower, it is higher than the threshold of significance of 5%.

Given that PAPm≥25mmHg is accompanied by LVEF> 50%, and the ratio E/E’<11, suggest Pre-capillary PH and exclude pulmonary venous hypertension.^3^ Therefore that PH is produced only by pulmonary arterial hypertension. The two major pathophysiological factors leading to increased pulmonary pressure are increased cardiac output and increased PVR.^2^-^5^ The values of these both factors become normal when achieving euthyroidism. The results are similar to our study.

Tachycardia, the increasing myocardial contractility (as effects of excess hormonal action over sympathetic nervous system^3^-^5^) and the decreased peripheral vascular resistance (as a result of overproduction of nitric oxide^3^,^4^) are the main factors leading to an increased cardiac output in hyperthyroid patients. As it was suggested, another mechanism for increasing the cardiac flow in hyperthyroid patients is the increase of total blood volume due to increased tubular reabsorption of sodium.^4^ There are other suggested mechanisms that produce pulmonary hypertension as the vascular endothelial dysfunction, caused by autoimmunity^4^ or by an increased metabolism of intrinsic pulmonary vasodilators or by a conversely decrease metabolism of intrinsic pulmonary vasoconstrictor.^4^,^5^ All these factors together lead to increase the pulmonary vascular resistance. In our study, the subgroup with PH, both cardiac output and PVR are significantly increased.

In the present study we identified new determinants for pulmonary hypertension as it follows: pretreatment period, age and systolic blood pressure. PAPs levels in hyperthyroidism decrease significantly with each decade of life. This fact suggests that the amplitude of hemodynamic changes in hyperthyroidism depends on vascular elasticity. After the age of 44, once the vascular rigidity increases PAPs and systolic and blood pressure start increasing. PAPs is also correlated with systemic pressure. This phenomenon is due to the action of the hormonal excess on sympathetic nervous system.

The method of polynomial regression according to the second degree equation “y=a+bx+cx^2^” (where y=PAPs, x=each determinant of PAPs separately and a, b and c are constant) improves coefficients of regression R^2^ and their level of significance compared to the method of linear regression that uses a first degree equation “y=a+bx” (where y=PAPs, x= each determinant of PAPs separately and a and b are constant).

The comparative analysis between the linear regression model and non-linear regression one (in our study being a parabola type) regarding the relation of determination of pulmonary pressure in hyperthyroidism shows that the polynomial model is better than the linear one in all situations because a) the coefficients of determination R^2^ are higher; b) residual sum of squares for polynomial regression are smaller than the residual sum of squares for linear regression highlighting the fact that the difference between the measured values and the values obtained through the polynomial model (values on the curve) are lower; c) ΔAIC has negative values and the more complicated model (parabola type one) is considered to be better.

Harvey Motulsky and Arthur Christopoulos^9^ concludes “Most models in biology are nonlinear, so many biologist use nonlinear regression more often than linear regression. Nonlinear regression is more general, as it can fit any model, including a linear one, to your data. Your choice of linear or nonlinear regression should be based on the model that makes the most sense for your data” in line with our opinion.

### Limitations of the study

PAPs has not been determined in an invasive manner, through catheterism, which is the method indicated for diagnosing HP in present guide, but HP was estimated through a echocardiographic method. Echocardiography is a less precise method through which one may overestimate a normal pulmonary pressure and may underestimate a severe pulmonary hypertension. We have to mention that the invasive measurement of PAPs in hyperthyroidism is not correct from an ethical point of view.

## CONCLUSIONS

The comparative analyses between linear and non-linear regression designed for creating a model of determining the pulmonary pressure in hyperthyroidism shows that the non-linear model is the most appropriate. The model of determining the pulmonary pressure in hyperthyroidism is a polynomial model represented by a second degree equation. The regression curve of a parabola type is matched with the clinical judgment.
